# Similarities in rotavirus vaccine viral shedding and immune responses in pairs of twins

**DOI:** 10.20407/fmj.2022-039

**Published:** 2023-05-09

**Authors:** Yasuko Enya, Hiroyuki Hiramatsu, Masaru Ihira, Ryota Suzuki, Yuki Higashimoto, Yusuke Funato, Kei Kozawa, Hiroki Miura, Masafumi Miyata, Yoshiki Kawamura, Takuma Ishihara, Koki Taniguchi, Satoshi Komoto, Tetsushi Yoshikawa

**Affiliations:** 1 Faculty of Clinical Engineering, Fujita Health University, School of Medical Sciences, Toyoake, Aichi, Japan; 2 Department of Clinical Pharmacy, Fujita Health University Hospital, Toyoake, Aichi, Japan; 3 Faculty of Clinical Science for Biological Monitoring, Fujita Health University, School of Medical Sciences, Toyoake, Aichi, Japan; 4 Faculty of Cellular and Molecular Biology, Fujita Health University, School of Medical Sciences, Toyoake, Aichi, Japan; 5 Department of Pediatrics, Fujita Health University, School of Medicine, Toyoake, Aichi, Japan; 6 Innovative and Clinical Research Promotion Center, Gifu University Hospital, Gifu, Gifu, Japan; 7 Department of Virology and Parasitology, Fujita Health University, School of Medicine, Toyoake, Aichi, Japan

**Keywords:** Rotavirus vaccine, Twins, Fecal viral shedding, Rotavirus IgG, Rotavirus IgA

## Abstract

**Objectives:**

Intestinal rotavirus (RV) vaccine replication and host immune response are suggested to be affected by several factors, including maternal antibodies, breastfeeding history, and gut microbiome, which are thought to be similar in pairs of twins. The aim of this study was to determine whether viral shedding from the fecal RV vaccine strain Rotarix^®^ (RV1) and IgG and IgA responses to RV show similarity in pairs of twins.

**Methods:**

Quantitative reverse transcription polymerase chain reaction specific to RV vaccine strain RV1 was used to monitor fecal RV1 viral shedding. RV IgG and IgA titers were measured using an in-house enzyme-linked immunosorbent assay. Fecal RV1 viral shedding and immune responses were compared between twins and singletons with mixed effects and fixed effects models.

**Results:**

A total of 347 stool and 54 blood samples were collected from four pairs of twins and twelve singletons during the observation period. Although the kinetics of fecal RV1 viral shedding and immune responses differed among vaccinated individuals, they appeared to be similar within twin pairs. RV shedding after the first dose (*P*=0.049) and RV IgG titers during the entire observation period (*P*=0.015) had a significantly better fit in the fixed effect model that assumed that twins have the same response versus the model that assumed that twins have a different response.

**Conclusions:**

The similarity of RV vaccine viral replication in intestine and host immune responses in twin pairs was demonstrated using statistical analysis.

## Introduction

Live attenuated rotavirus (RV) vaccines can decrease RV-related morbidity and mortality,^1^ and the World Health Organization recommends RV vaccination for all children.^2^ The two most common live attenuated oral RV vaccines used globally are Rotarix^®^ (RV1, GlaxoSmithKline Biologicals, Rixensart, Belgium) and RotaTeq^®^ (RV5, Merck & Co., New Jersey, USA). RV vaccines are highly effective in countries with low mortality but are insufficient in those with medium to high levels of mortality.^3^ These differences can be explained by several mechanisms, including high titers of RV-specific IgA antibodies derived from breast milk^4^ and of maternal RV-specific IgG antibodies,^5^ variations in histo-blood group antigens that are RV genotype-dependent receptors,^6^ and differences in the gut microbiome.^7^

Our institute conducted a prospective study to assess the safety of RV vaccines in neonatal intensive care units by monitoring fecal vaccine viral shedding. We demonstrated that RV vaccination can be performed safely in neonatal intensive care units with contact precaution measures.^8^ A cohort study to assess whether there is an association between RV vaccine viral shedding in stool samples and host immune responses is ongoing. Fecal RV vaccine viral shedding can vary by vaccine recipient as demonstrated in a clinical study using a RV5-specific quantitative revere transcription polymerase chain reaction (qRT-PCR).^9^ As research progressed, the number of twin pairs enrolled in this study increased gradually, and fecal RV vaccine viral shedding and host immune responses appeared to be similar within those twin pairs. Levels of maternal antibodies and IgA antibodies derived from breast milk appeared to be similar within the twin pairs. In addition, host factors, including the composition of the gut microbiome, were also similar within the twin pairs.^10–12^ As mentioned above, these factors have been associated with host immune responses against RV vaccines, and fecal RV vaccine viral shedding and immune responses are likely to be similar within twin pairs. Therefore, the aim of this study was to determine whether fecal RV1 viral shedding and RV IgG and IgA responses are similar in twin pairs.

## Methods

### Study design

To examine the association between fecal RV vaccine viral shedding and vaccine-induced immune responses, we conducted a prospective cohort study of infants admitted to the neonatal intensive care unit of Fujita Health University Hospital. Twenty vaccinated infants (four pairs of twins and twelve singletons) born between November 2018 and June 2020 were enrolled in this study. This study was approved by the review board of our university (approval no. HM17-037). The infants’ guardians consented to their participation.

### Sample collection

To monitor RV1 vaccine strain viral shedding, stool samples were collected daily from day 0 (i.e., day of vaccination) until day 8 after the second of two vaccination doses. A 1-mL blood sample was obtained before the first vaccine dose, at 1 month after the first dose, and at 1 month after the second dose.

### Serum RV IgG and IgA measurement

An enzyme-linked immunosorbent assay (ELISA) was performed to measure serum anti-RV IgG and IgA titers as previously described with modifications.^13^ The ELISA plate was coated with 25 μL of purified human rotavirus strain Wa virions (1 μg/mL) overnight at 4°C. After blocking and washing with Tris-buffered saline containing Tween 20, 25 μL of diluted samples (1:10–1:10^6^) were added to each well and incubated at room temperature for 1 h. After washing, detection of anti-RV IgG or IgA was performed at room temperature over 1 h using 25 μL of peroxidase-labeled goat anti-human IgG (0.1 μg/mL) or anti-human IgA (0.5 μg/mL) (Kirkegaard & Perry Laboratories, Gaithersburg, MD, USA). The antigen–antibody complexes were detected using 1-Step Ultra TMB-ELISA Substrate Solution (Thermo Fisher Scientific, Waltham, MA, USA) according to the manufacturer’s instructions. Five RV antibody-negative serum samples were used to determine cutoff values. The cutoff optical density value for negative anti-RV IgG and IgA was defined as the mean+three standard deviations of negative samples (RV IgG, 0.20; RV IgA, 0.20). Serum IgG and IgA titers were calculated as the highest dilution that gave a mean optical density greater than the cutoff value. A four-fold or higher increase in IgG or IgA titers during the first or second dose samples compared with the before samples was defined as a significant increase.

### RV1 qRT-PCR

For each stool sample, 1 mL of a 10% solution was prepared in physiological saline solution. Each suspension was clarified by centrifugation for 20 min at 4,000 *g*. Next, 140 μL of the supernatant was used for RNA extraction. using the QIAamp viral RNA MiniKit (QIAGEN, Chatsworth, CA, USA). RV1-specific qPCR was used to monitor fecal RV1 viral shedding.^14^

### Statistical analysis

Baseline characteristics are presented as the medians and interquartile range (IQR) for continuous variables. For categorical variables, frequency and proportion are presented. To assess the similarity in RV IgG and IgA responses and viral shedding in twins, the goodness of fit models were compared. One model (twin model) was a mixed-effects model in which random effects were incorporated with the assumption that the data obtained from twins are correlated. The other model (without twin model) was a fixed effects model in which data from twins was assumed to be independent. The similarity within a twin pair was examined during two periods: (1) before and after the first dose of vaccination and (2) before the first dose of vaccination and after the second dose of vaccination. The goodness of fit of the models was assessed using Akaike information criterion (AIC).^15^ When the goodness of fit of the model including random effects was high (i.e., the AIC value was small) and significant differences could be detected with the likelihood ratio test, the twins were considered to be similar. All analyses were performed using R software version 4.1.1 (R Project for Statistical Computing, Vienna, Austria).

## Results

A total of 347 stool specimens were collected from 20 vaccinated infants as described above during the observation period. There were 143 samples collected from twins with 72 samples collected before and after the first dose and 71 samples collected before and after the second dose. One twin could not be sampled at day 8 after the second dose. There were 204 samples collected from singletons, of which 105 were samples collected before and after the first dose (three singletons failed to provide a single stool sample) and 99 samples were collected before and after the second dose (nine singletons failed to provide a single stool sample). A blood sample could not be collected from one pair of twins (pair D) and four singletons at 1 month after the second vaccination. All other blood samples (54 samples) were serially collected as per the study protocol.

The baseline characteristics of the twins and singletons are summarized in Table 1. Birth weight was significantly lower in singletons than that in twins (*P*=0.023). Generally, singleton infants have a higher birth weight than twin infants do. However, in this study, five singletons had fetal growth restriction caused by several complications such as excess torsion of the cord, subchorionic hematoma, pregnancy-induced hypertension, and single umbilical artery, whereas none of the twins had fetal growth restriction. Thus, the body weight of the singletons was significantly lower than that of the twins. All infants were delivered by cesarean section. Pair A consisted of monochorionic monoamniotic twins, pair B consisted of monochorionic diamniotic twins, and pairs C and D consisted of dichorionic diamniotic twins. There were no statistical differences in feeding procedures between twins and singletons. All infants received two doses of the RV1 vaccine.

**Figure 1:**
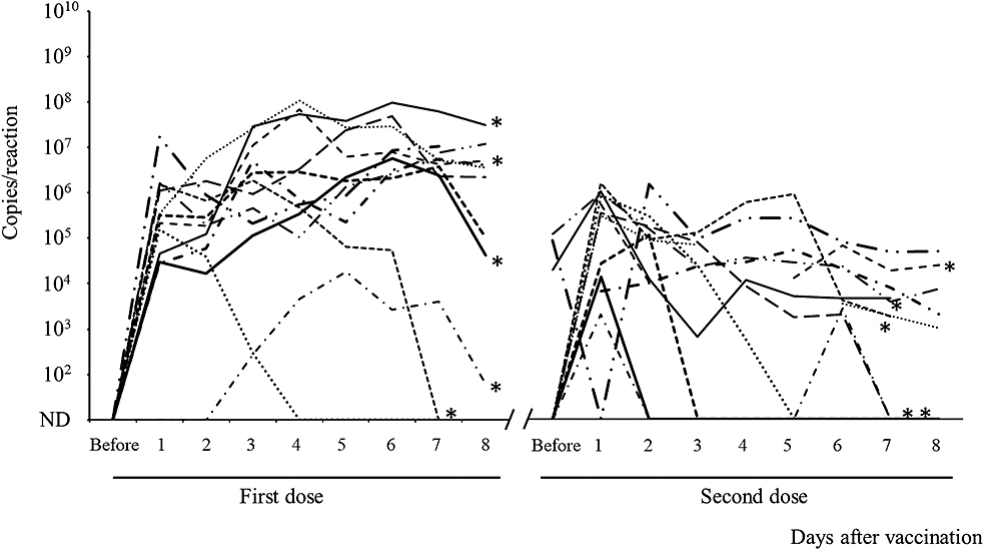
Kinetics of fecal Rotarix^®^ (RV1) viral shedding from 12 singletons after the first and second doses of the RV1 vaccine Stool samples were collected before and 8 days after the first and second vaccinations, respectively. Fecal RV1 RNA was measured using RV1-specific real-time reverse transcription polymerase chain reaction. The differently shaped line indicates the RV1 RNA shedding in the stools of each infant. The five fetal growth restriction cases are marked with an asterisk. ND, not detected.

The fecal RV1 RNA shedding from the singletons for 8 days after the first and second doses of the vaccine is shown in Figure 1. Although three singletons had low levels of RV1 RNA shedding for a short duration, the remaining nine singletons had persistent RV1 RNA shedding during the 8 days after the first vaccine dose. RV1 RNA was detected in three stool samples obtained before the second vaccine dose. The kinetics of RV vaccine viral shedding varied among vaccine recipients, including infants with fetal growth restriction. The peak viral RNA load appeared to be lower after the second dose than after the first dose, and the kinetics of fecal RV1 RNA shedding after the first and second doses differed by infant.

**Figure 2:**
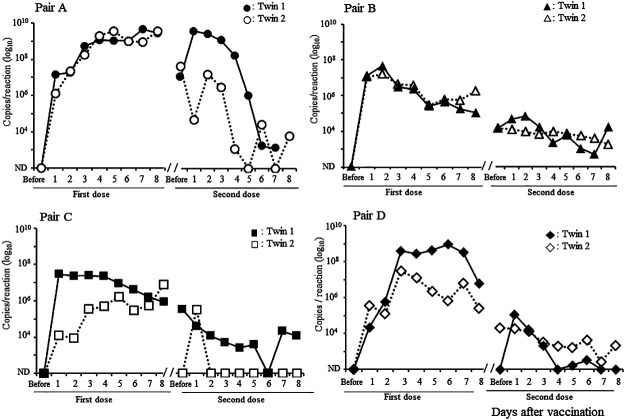
Kinetics of fecal Rotarix^®^ (RV1) viral shedding of the 4 twin pairs after the first and second doses Each pair is represented by a different shape. Black and white symbols represent the two infants in the pair. Circle, pair A; triangle, pair B; square, pair C; diamond, pair D. ND, not detected.

The fecal RV1 RNA shedding of the twins is shown in Figure 2. The kinetics of this shedding was similar for each pair of twins for both the first and second doses. RV1 RNA levels in the stool increased quickly soon after the first vaccination and continued to gradually increase until day 8 in pair A, whereas in pair B, these increased quickly and then gradually decreased. In contrast to pair A, pair B had relatively low RV1 RNA levels before the second vaccination, which gradually decreased until 8 days after the second vaccination. In pairs C and D, as in pair B, the excretion of RV1 RNA increased and then gradually decreased after the first dose. Pairs C and D had relatively low copy numbers of RV1 RNA that gradually decreased after the second dose.

**Figure 3:**
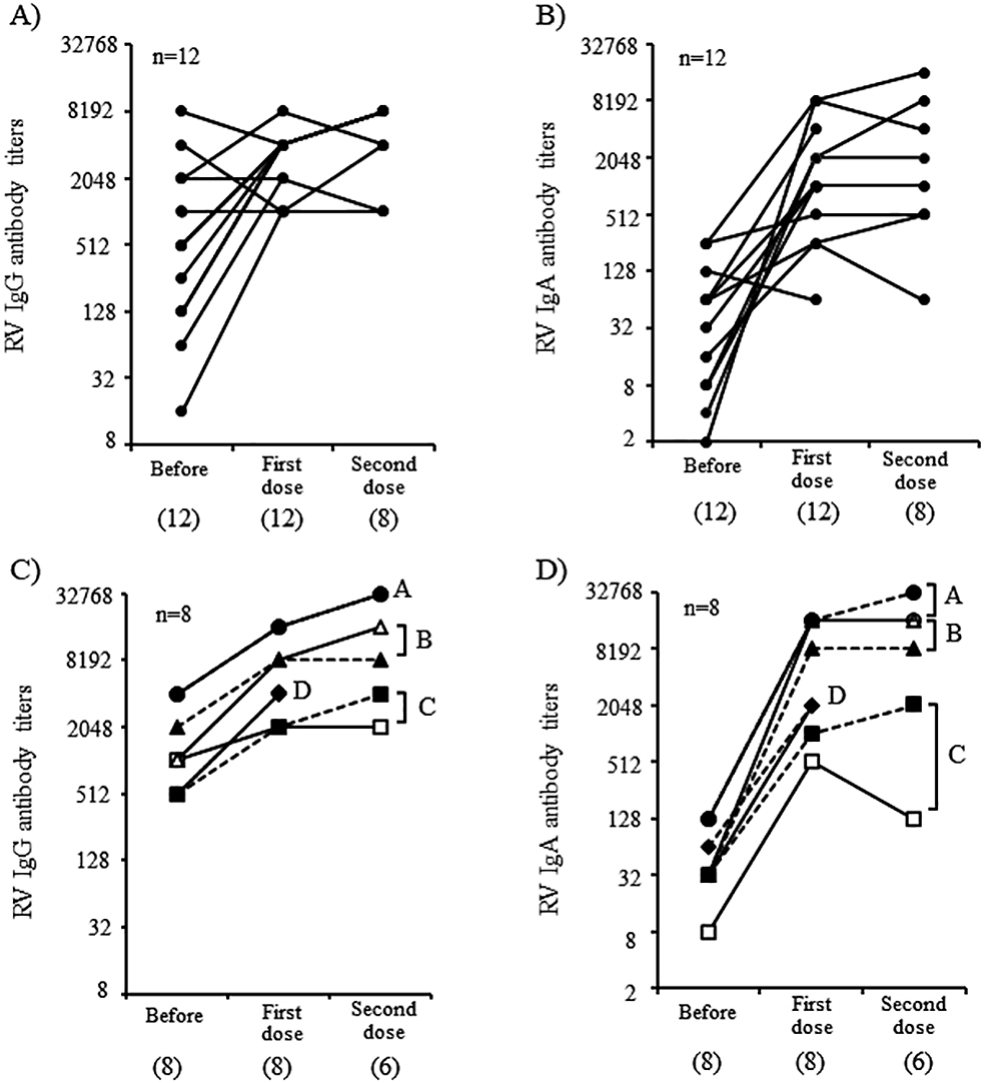
Serum rotavirus (RV) IgG and IgA responses in singletons and twins during the observation period Kinetics of RV IgG and IgA antibody titers in the 12 singletons are shown in (A) and (B), respectively. Kinetics of RV IgG and IgA antibody titers in the four twin pairs are shown in (C) and (D), respectively. The antibody titers of each twin pair are represented by a different shape. Black and white symbols represent the two infants in the pair. Circle, pair A; triangle, pair B; square, pair C; diamond, pair D. The IgG antibody titers of twin pairs A and D (C) and IgA titers after the first dose in twin pair D (D) were the same. Information for each infant and their antibody titers are presented in the supplemental Table.

The kinetics of RV IgG and IgA antibodies in singletons and twins are shown in Figure 3. A significant increase in RV IgG and IgA antibody titers was observed in 14 of 20 (70.0%) infants and 18 of 20 (90%) infants, respectively, over the entire observation period. The RV IgG (Figure 3A) and IgA (Figure 3B) antibody titers (Supplemental table) significantly increased in 7 of 12 (58.3%) singletons and 10 of 12 (83.3%) singletons, respectively, after the first dose of vaccine, respectively. Although only eight serum samples were collected after the second dose of vaccine was available for serological analysis, most infants did not exhibit a significant booster effect, which was probably due to a sufficient increase in both IgG and IgA antibody titers after the first vaccine dose.

The kinetics of RV IgG (Figure 3C) and IgA (Figure 3D) antibody titers were similar in each pair of twins. A significant increase in these titers was observed after the first dose of RV1 vaccine except with one twin of Pair C, whose IgG titers did not increase after the second dose (indicated with open squares). All four pairs of twins had significant increases in RV IgA antibody titers after the first dose. For one twin of pair C (indicated with open squares in Figure 3), IgA titers increased significantly but subsequently decreased after the second dose.

AIC values were used to compare a model that assumed correlation between twins and a model that assumed independence between twins to determine which model had a better fit to the data. The AIC value for RV viral shedding was smaller in the twin model compared to the without twin model for the first dose. Similarly, the AIC value for the twin model was smaller for RV IgG and IgA titers over the entire observation period. There was a significant difference in AIC values between the models for viral shedding (*P*=0.049), there was no significant differences in AIC values for RV IgG (*P*=0.057) or IgA titers (*P*=0.207) based on the likelihood ratio test for the first dose models. Conversely, no significant differences in AIC values between either model for viral shedding (*P*=0.248) or RV IgA antibody titers (*P*=0.058) were observed over the entire observation period, but there was a significant difference in AIC values for RV IgG titers (*P*=0.015) (Table 2).

## Discussion

Our previous studies suggested that the kinetics of RV vaccine viral shedding vary among vaccine recipients.^8,9^ Herein, as similarly reported,^14^ study participants exhibited variable fecal RV shedding after vaccination, with several quickly decreasing in fecal RV1 viral shedding while others had prolonged fecal shedding after the first dose. In general, viral RNA loads in stool samples appeared to be lower after the second dose compared with that after the first dose. The kinetics of fecal RV1 viral shedding that showed lower viral shedding after the second dose were also consistent with previous studies,^16^ suggesting that the host immune response induced by the first vaccine dose suppressed RV1 replication after the second dose.

We found that the kinetics of fecal RV1 RNA shedding after the first and second vaccine doses were similar within each pair of twins (Figure 2) and that the IgA responses after RV vaccination were also similar within each pair of twins (Figure 3C and D). These findings suggest that twin siblings share factors that help control RV vaccine replication in intestinal tissues and host immune responses. The goodness of fit of the mixed effects and fixed effects models were compared to determine whether fecal RV1 viral shedding and immune responses were similar between twins. The better fit of the mixed effects model suggests that viral shedding and the subsequent immune response is correlated among twins. The likelihood ratio test is used to statistically analyze the goodness of fit of the two models. For the first dose, the model of RV shedding assumed that the twins have the same response and had a significantly better fit than the model assuming the twins have a different response, and thereby indicates any similarity in viral replication within twin pairs (*P*=0.049). However, the similarity in RV1 viral shedding in twins was not confirmed during the period from prevaccination to after the second RV vaccine dose. The model of RV IgG responses that assumed twins have the same response had a significantly better fit than the model that assumed twins have a different response, indicating a similarity in IgG responses from prevaccination to post-second vaccination within a twin pair (*P*=0.015). Therefore, these data show that RV1 viral shedding after the first vaccination and the IgG response before and after two vaccine doses were statistically similar within twin pairs. Meanwhile, the RV IgA antibody responses during the entire observation period tended to be similar in twins, although this was not significant different (*P*=0.058). Increasing the number of twins in a future study may uncover a significant relationship for the RV IgG antibody responses. Therefore, this is the first report showing significant similarities in RV vaccine viral replication and host immune response within twin pairs.

Feeding was the same in each pair of twins but was diverse across singletons. Although the gut microbiome was not analyzed in this study, it has been suggested that the gut microbiomes in pairs of twins have greater similarities than those between two randomly selected infants. Delivery and feeding, which are generally identical within twin pairs, may influence the composition of the infant gut microbiome.^17–20^ In addition, the gut microbiome is thought to be involved in the development of both innate and adaptive immune training during early infancy.^21^ Many factors are involved in vaccine viral replication and host immune responses induced by RV vaccination, and it would be challenging to elucidate the mechanisms responsible for the similarities in RV vaccine viral replication and host immune responses between twins. However, understanding the precise mechanism underlying similar RV vaccine viral shedding and immune responses in pairs of twins may be valuable in improving the efficacy of RV vaccines in countries with medium- and high-mortality rates.

The complexity of the various factors involved in fecal RV shedding and host-immune responses among singletons impedes understanding the precise mechanisms involved in viral replication, vaccination, and host immune responses within a singleton cohort. However, studies targeting twins are considered helpful to simplify these analyses because of the similarity of their backgrounds. Indeed, although pairs B and D were breastfed, the RV IgG and IgA levels increased significantly after the second dose in these two pairs, despite slightly lower fecal RV1 viral shedding than in pair A. Several human studies^5^ and animal model experiments^22^ have suggested that maternal or transferred RV IgG antibodies suppress RV vaccine viral replication and weaken the host immune response. In this study, although high levels of maternal RV IgG antibody titers were detected in sera obtained before vaccination from pair A, both twins in pair A had more fecal RV1 viral shedding than that in the three other pairs. In addition, the maternal RV IgG antibody titers were relatively low in pairs C and D, but we did not observe high RV1 viral shedding in these pairs. Thus, although RV vaccine viral replication and host immune response were similar in each pair of twins in this study, the levels of viral replication and immune response differed from theoretical values based on previous studies.

We only analyzed four pairs of twins were in this study, and it is difficult to establish an association between fecal RV vaccine viral shedding and immune responses, even if the similarities between the twins were statistically confirmed. However, high levels of RV vaccine viral shedding were observed in pair A, and the highest RV IgG and IgA antibody titers after the second dose of RV vaccine were observed in these two infants. In contrast to pair A, in one twin of pair C (indicated with open squares), RV IgA antibody titers after the second dose of the RV vaccine were low (×128) (indicated with open squares in Figure 3D). Furthermore, the RV IgG antibody titer (×2048) was relatively low in serum collected after the second dose (indicated with open squares in Figure 3C); similarly, levels of vaccine viral shedding were also relatively low in this infant. In addition, singletons with low levels of RV1 viral shedding after the first dose had weak IgG and IgA immune responses. A reduced RV vaccine viral replication has been associated with a weaker immune response,^23^ and a low replication of RV1 in twin two of pair C may have contributed to a lower level of immune responses induced by vaccination. RV IgA antibody titers after the second dose are significantly correlated with RV vaccine efficacy.^24^ Therefore, to improve RV vaccine efficacy, it will be important to develop measures to promote RV vaccine viral replication in intestinal tissue. In addition, precise determination of how to evaluate viral replication is important. Several cases with positive RV1 shedding before the second vaccination dose appeared to have long-term RV1 viral replication. Therefore, the duration of the RV1 shedding may be useful for assessing vaccine virus replication in intestinal tissue.

## Conclusions

We compared random and fixed effects models and demonstrated significant similarities in RV vaccine viral replication and immune responses in twins. Since many of the factors associated with RV vaccine viral replication and host immune responses will be similar between twin pairs, these findings are reasonable. Although several factors such as maternal antibody titers and feeding procedure have already been analyzed, the influence of the intestinal microbiome remains unclear in this study. Analysis of gut microbiomes is currently underway in this cohort to elucidate the role of the microbiome in influencing RV vaccine viral replication and host immune responses.

## Figures and Tables

**Table1:** Baseline characteristics of four pairs of twins and 12 singletons

Characteristic	Twins (n=8; four pairs)	Singletons (n=12)	*P*-value
Gender			
Male/Female (%)	Pair A, B: Female, Female Pair C: Male, Female Pair D: Male, Male	8 (66.7)/4 (33.3)	0.362
Birth weight, g			
Median (IQR)	1237 (1200–1319)	1038 (966–1147)	0.023
Gestational age, wk			
Median (IQR)	30.5 (30.3–30.9)	28.8 (27.1–30.4)	0.097
Chronological age^a^, wk			
Median (IQR)	9.1 (9.0–9.5)	11.6 (10.8–14.0)	0.132
Mode of delivery			
Vaginal delivery	0	0	—
Cesarean section	8	12	
Feeding	Mix-fed: Pair A, C	Mix-fed: 4	
Formula/Breastfeeding/Mix	Breast-fed: Pair B, D	Breast-fed: 7	0.795
		Formula-fed: 1	

^a^ At the time of the first dose of vaccination. IQR: Interquartile range; wk: Weeks

**Table2:** Comparison of the goodness of fit between a model that considers that twins have the same response and a model that considers that twins have a different response

Variable	AIC value		*P* value
	^a^Twin model	^b^Personally allocated model (without twin)	
First dose			
RV shedding	782.09	783.93	0.049
RV IgG	134.71	136.34	0.057
RV IgA	147.81	147.4	0.207
Entire observation period			
RV shedding	1757.7	1757.0	0.248
RV IgG	170.19	174.06	0.015
RV IgA	201.46	203.06	0.058

^a^ Twin model: a model that considers twins have the same response (same person). ^b^ Personally allocated model (without twin): Twins have a different response. AIC: Akaike information criterion; RV: rotavirus.
