# Supplementary material for: Similarities in rotavirus vaccine viral shedding and immune responses in pairs of twins
Source: Fujita Med J. 2023 May 9;9(3):253–8. doi: 10.20407/fmj.2022-039 (PMC10405898; doi:10.20407/fmj.2022-039)
Supplement: Supplementary file 1 — Supplementary Table [file fmj-9-253-s001.pdf]

Supplemental Table. Detail of antibody titers in each case.

No. of cases	Singleton /Twin	RV-IgG				RV-IgA			
		Before	First dose	Second dose	Significant Increase [b]	Before	First dose	Second dose	Significant Increase [b]
1	Twin A	4096	16384	32768	Yes	128	16384	16384	Yes
2	Twin A	4096	16384	32768	Yes	128	16384	32768	Yes
3	Twin B	1024	8192	16384	Yes	32	16384	16384	Yes
4	Twin B	2048	8192	8192	Yes	32	8192	8192	Yes
5	Twin C	512	2048	4096	Yes	32	1024	2048	Yes
6	Twin C	1024	2048	2048	No	8	512	128	Yes
7	Twin D	512	4096	NT	Yes	32	2048	NT	Yes
8	Twin D	512	4096	NT	Yes	64	2048	NT	Yes
9	Singleton	1024	1024	1024	No	256	512	512	No
10	Singleton	2048	8192	4096	Yes	256	8192	4096	Yes
11	Singleton	256	4096	8192	Yes	8	2048	8192	Yes
12	Singleton	128	4096	8192	Yes	4	2048	2048	Yes
13 [a]	Singleton	16	1024	4096	Yes	8	1024	1024	Yes
14 [a]	Singleton	64	2048	1024	Yes	16	256	64	Yes
15 [a]	Singleton	512	4096	8192	Yes	4	8192	16384	Yes
16	Singleton	8192	4096	8192	No	64	256	512	Yes
17 [a]	Singleton	4096	1024	NT	No	128	64	NT	No
18 [a]	Singleton	1024	1024	NT	No	32	1024	NT	Yes
19	Singleton	2048	2048	NT	No	64	4096	NT	Yes
20	Singleton	128	4096	NT	Yes	64	1024	NT	Yes

[a] Fetal growth restriction cases.

[b] A four-fold or higher increase in IgG or IgA titers during the first or second dose samples compared with the before samples was defined as a significant increase. NT: Not tested
